# Mesenchymal stromal cells and paracrine modulation of neuroplasticity in the central nervous system

**DOI:** 10.17179/excli2026-9599

**Published:** 2026-08-24

**Authors:** Sacha Krolow e Silva, Iris Virgínia Rondanine Sanches, Juliana Soares Lara de Lima, Luciana Le Sueur-Maluf

**Affiliations:** 1 Specialization Course in Neuroscience, Universidade Federal de São Paulo (UNIFESP), Santos, São Paulo, Brazil; 2 Graduate Program in Biological Sciences: Biochemistry, Universidade Federal do Rio Grande do Sul (UFRGS), Porto Alegre, Rio Grande do Sul, Brazil; 3 Instituto Superior de Educação União (UNISED), São Paulo, São Paulo, Brazil; 4 Graduate Program in Neurology/Neuroscience, Universidade Federal de São Paulo (UNIFESP), São Paulo, São Paulo, Brazil; 5 Department of Biosciences, Universidade Federal de São Paulo (UNIFESP), Santos, São Paulo, Brazil

**Keywords:** mesenchymal stromal cells, extracellular vesicles, paracrine signaling, neuroplasticity, glymphatic system, central nervous system disorders

## Abstract

Mesenchymal stromal cells (MSCs) have been widely investigated in regenerative medicine because of their immunomodulatory, anti-inflammatory, and reparative properties. Current evidence indicates that many of their effects are mediated by paracrine and intercellular mechanisms rather than sustained engraftment or direct neural replacement. This narrative review synthesizes how MSCs and MSC-derived extracellular vesicles (MSC-EVs) may modulate neuroplasticity in the central nervous system (CNS). MSC-mediated effects include trophic and immunomodulatory signaling, mitochondrial transfer via tunneling nanotubes (TNTs), efferocytosis of apoptotic MSCs by host phagocytes, blood-brain barrier stabilization, and secondary modulation of glymphatic and interstitial fluid clearance. MSC-EVs additionally deliver proteins, lipids, microRNAs, and long non-coding RNAs that regulate inflammatory and reparative gene-expression programs through well-supported post-transcriptional and emerging epigenetic mechanisms. These processes may promote neurite remodeling, angiogenesis, mitochondrial recovery, and attenuation of neuroinflammation, and may support the clearance of neurotoxic proteins. The evidence is organized into five CNS disorder macrocategories defined by shared pathophysiological processes and overlapping therapeutic targets: cerebrovascular and ischemic/hemorrhagic disorders; neurodegenerative proteinopathies and related disorders; neurotrauma and acute lesions; autoimmune, inflammatory, and emerging disorders; and retinal diseases as CNS-related sensory disorders. Although substantial preclinical evidence and encouraging early clinical signals support continued investigation, adequately powered confirmatory trials are still needed to establish whether MSC-based thera5pies can produce durable disease-modifying effects in neurodegenerative disorders. Translation is further limited by variability in cell sources, manufacturing procedures, doses, delivery routes, potency assays, and patient stratification by disease stage and biological phenotype. A cohesive translational roadmap should therefore combine Good Manufacturing Practice (GMP)-compliant production and mechanism-relevant potency testing with the 2025 International Society for Cell & Gene Therapy (ISCT) reporting framework. Finally, engineered MSC-EVs with modified surfaces or defined cargo may enable more targeted and controllable cell-free strategies for CNS repair.

See also the graphical abstract(Fig. 1).

## Abbreviations

otz425**Aβ**, amyloid-beta; **ac-tau**, acetylated tau; **AD**, Alzheimer's disease; **AD-MSCs**, adipose tissue-derived mesenchymal stromal cells; **ALS**, amyotrophic lateral sclerosis; **AQP4**, aquaporin-4; **ASOs**, antisense oligonucleotides; **ATP**, adenosine triphosphate; **BACE1**, beta-site amyloid precursor protein-cleaving enzyme 1; **BBB**, blood-brain barrier; **BDNF**, brain-derived neurotrophic factor; **BM-MSCs**, bone marrow-derived mesenchymal stromal cells; **BRB**, blood-retinal barrier; **ceRNA**, competing endogenous RNA; **CNS**, central nervous system; **CSF**, cerebrospinal fluid; **CXCR4**, C-X-C chemokine receptor type 4; **DMN**, default mode network; **EDSS**, Expanded Disability Status Scale; **EVs**, extracellular vesicles; **FDA**, U.S. Food and Drug Administration; **fMRI**, functional magnetic resonance imaging; **GDNF**, glial cell line-derived neurotrophic factor; **GMP**, Good Manufacturing Practice; **HDAC4**, histone deacetylase 4; **HIE**, hypoxic-ischemic encephalopathy; **HIF-1'e1**, hypoxia-inducible factor 1-alpha; **hPSCs**, human pluripotent stem cells; **I/R**, ischemia/reperfusion; **ICH**, intracerebral hemorrhage; **IDE**, insulin-degrading enzyme; **IDO**, indoleamine 2,3-dioxygenase; **IFN-γ**, interferon-gamma; **IL-1β**, interleukin-1 beta; **IL-4**, interleukin-4; **IL-6**, interleukin-6; **IL-10**, interleukin-10; **IL-18**, interleukin-18; **IRAK1**, interleukin-1 receptor-associated kinase 1; **ISCT**, International Society for Cell & Gene Therapy; **ISF**, interstitial fluid; **iMSCs**, induced pluripotent stem cell-derived mesenchymal stromal cells; **iPSCs**, induced pluripotent stem cells; **IVH**, intraventricular hemorrhage; **LC3-II**, microtubule-associated protein 1A/1B-light chain 3-II; **lncRNA**, long non-coding RNA; **MAPK**, mitogen-activated protein kinase; **miRNA**, microRNA; **miR**, mature microRNA designation; **Miro1**, mitochondrial Rho GTPase 1; **MMP-2/MMP-9**, matrix metalloproteinase-2/matrix metalloproteinase-9; **mRNA**, messenger RNA; **MRI**, magnetic resonance imaging; **MS**, multiple sclerosis; **MSC-EVs**, mesenchymal stromal cell-derived extracellular vesicles; **MSCs**, mesenchymal stromal cells; **NAD+**, nicotinamide adenine dinucleotide; **NAMPT**, nicotinamide phosphoribosyltransferase; **NEP**, neprilysin; **NF-κB**, nuclear factor kappa B; **NIHSS**, National Institutes of Health Stroke Scale; **NLRP3**, NOD-, LRR- and pyrin domain-containing protein 3; **OPC**, oligodendrocyte precursor cell; **PD**, Parkinson's disease; **PGE2**, prostaglandin E2; **PI3K/Akt/mTOR**, phosphoinositide 3-kinase/protein kinase B/mechanistic target of rapamycin; **PTEN**, phosphatase and tensin homolog; **RhoA**, Ras homolog family member A; **ROS**, reactive oxygen species; **rTBI**, repetitive traumatic brain injury; **RVG**, rabies virus glycoprotein; **SCI**, spinal cord injury; **SDF-1**, stromal cell-derived factor 1; **siRNA**, small interfering RNA; **SIRT1**, sirtuin 1; **SMI**, severe mental illness; **SNCA**8 , synuclein alpha gene; **Sox9**, SRY-box transcription factor 9; **TBI**, traumatic brain injury; **TGF-β**, transforming growth factor-beta; **Th1**, T helper 1 cell; **Th17**, T helper 17 cell; **TLR4**, toll-like receptor 4; **TNF-α**, tumor necrosis factor-alpha; **TNTs**, tunneling nanotubes; **TRAF6**, TNF receptor-associated factor 6; **Treg**, regulatory T cell; **TSG-6**, tumor necrosis factor-stimulated gene-6; **UC-MSCs**, umbilical cord-derived mesenchymal stromal cells; **VEGF**, vascular endothelial growth factor

## 1. Introduction

Neuroplasticity, or brain plasticity, refers to the ability of the nervous system to reorganize its structural and functional connections in response to stimuli, injury, and experience, thereby supporting adaptation to the environment, learning, and functional recovery (Mateos-Aparicio and Rodríguez-Moreno, 2019[45]). For decades, the adult brain was regarded as a largely static structure with limited regenerative capacity. This view was progressively challenged by evidence demonstrating adult neurogenesis in specific brain regions, particularly the hippocampus (La Rosa et al., 2020[33]; Owji and Shoja, 2020[53]). Although neuroplasticity is primarily adaptive, dysregulated plastic responses may also contribute to the pathophysiology of neurological and psychiatric disorders (Sweatt, 2016[70]).

Neurodegenerative diseases, such as Alzheimer's disease and Parkinson's disease, acute central nervous system (CNS) injuries such as stroke, and severe mental illnesses (SMI), including schizophrenia and bipolar disorder, remain major challenges in contemporary medicine. Although heterogeneous in etiology and clinical presentation, these conditions involve convergent pathological processes that can impair neural plasticity and repair, including synaptic dysfunction, neuroinflammation, mitochondrial impairment, cellular vulnerability, and, in neurodegenerative proteinopathies, accumulation of misfolded or aggregation-prone proteins (Wilson et al., 2023[82]; Zhang et al., 2020[91]). Current therapeutic approaches are predominantly symptomatic and often fail to prevent disease progression, underscoring the need for new strategies that can modulate the neural microenvironment and support repair mechanisms.

Stem cell-based approaches have been investigated as potential therapeutic strategies for these conditions. Human pluripotent stem cells (hPSCs) offer broad differentiation potential. Among them, induced pluripotent stem cells (iPSCs), first generated from adult human fibroblasts using defined transcription factors, have become important platforms for disease modeling and regenerative research (Takahashi et al., 2007[71]). However, their clinical application has been limited by ethical considerations, genetic instability, tumorigenic risk, and concerns about immune compatibility (Lo and Parham, 2009[41]; Qiao et al., 2020[56]). More recently, iPSC technology has been used to derive induced pluripotent stem cell-derived mesenchymal stromal cells (iMSCs), providing a potentially scalable and more homogeneous source that may help overcome donor-to-donor variability commonly observed in primary tissue-derived mesenchymal stromal cells (Buitrago et al., 2024[7]; Lu and Allickson, 2025[43]).

The clinical feasibility of mesenchymal stromal cell (MSC)-based therapy has been reinforced by the 2024 approval of remestemcel-L-rknd (Ryoncil) by the U.S. Food and Drug Administration (FDA) for steroid-refractory acute graft-versus-host disease in pediatric patients aged 2 months and older (Lu and Allickson, 2025[43]; Wiest, 2025[81]). However, translating MSC-based approaches to CNS disorders remains investigational and requires disease-specific validation. Although these cells have historically been referred to as “stem cells,” current scientific and regulatory consensus increasingly recommends the term “stromal cells,” reflecting the view that their main therapeutic effects are mediated by microenvironmental modulation rather than by direct differentiation or long-term tissue replacement (Renesme et al., 2025[61]; Wiest, 2025[81]).

Instead of acting primarily through direct cell replacement, MSCs are thought to influence neuroplasticity through multiple paracrine and cell-mediated mechanisms. These include: (1) the secretion of extracellular vesicles (EVs) carrying regulatory microRNAs, such as miR-133b and miR-21, which may contribute to neurite remodeling and angiogenesis (Nasirishargh et al., 2021[50]; Xu et al., 2025[87]); (2) mitochondrial transfer to injured neural cells, which may support cellular bioenergetics (Mukkala et al., 2023[48]); and (3) efferocytosis, in which apoptotic MSCs are engulfed by host immune cells, potentially inducing sustained anti-inflammatory responses (Hoseinzadeh et al., 2025[28]). Beyond these direct mechanisms, MSC-based interventions may also modulate functional CNS clearance pathways, including glymphatic activity, by affecting astrocytic aquaporin-4 organization, blood-brain barrier integrity, neuroinflammatory signaling, and interstitial fluid clearance (Li et al., 2026[37]; Zhao et al., 2026[92]).

This narrative review synthesizes current evidence on the therapeutic potential of MSCs and their derivatives in the CNS, with emphasis on their paracrine modulation of neuroplasticity. The novelty of this review lies in its unified framework, which integrates mechanisms mediated by MSCs and their extracellular vesicles across five CNS macrocategories, organized by shared pathophysiological mechanisms and overlapping therapeutic targets. Although etiologically distinct, these conditions converge on chronic neuroinflammation, neural barrier dysfunction, and impaired neuroplasticity, supporting a conceptual shift from isolated disease-specific approaches toward broader biological repair strategies. Finally, to address the gap between preclinical evidence and clinical translation, we discuss manufacturing and regulatory challenges, including Good Manufacturing Practice (GMP)-related requirements, and highlight the relevance of the 2025 International Society for Cell & Gene Therapy (ISCT) clinical reporting guidelines for improving methodological standardization and reproducibility. The conceptual framework and principal mechanisms discussed in this review are summarized in Figure 1(Fig. 1).

## 2. Methods

We conducted a narrative literature review to synthesize experimental, translational, and clinical evidence on the potential role of MSCs and MSC-derived products in modulating neuroplasticity and supporting repair mechanisms in CNS disorders. The literature search was conducted in PubMed, SciELO, Scopus, Web of Science, and Google Scholar, considering publications in English and Portuguese. The search strategy used different combinations of free-text keywords and Medical Subject Headings (MeSH terms), including “mesenchymal stromal cells,” “mesenchymal stem cells,” “extracellular vesicles,” “exosomes,” “post-transcriptional and epigenetic modulation,” “neuroplasticity,” “paracrine signaling,” “neuroinflammation,” “mitochondrial transfer,” “efferocytosis,” “glymphatic system,” “blood-brain barrier,” “central nervous system,” “neurodegenerative diseases,” “Alzheimer's disease,” “Parkinson's disease,” “amyotrophic lateral sclerosis,” “stroke,” “intracerebral hemorrhage,” “traumatic brain injury,” “spinal cord injury,” “multiple sclerosis,” and “retinal diseases.”

Because this is a narrative review focused on the conceptual evolution, mechanisms of action, and translational relevance of MSC-based approaches, no date restriction was applied. This strategy enabled the inclusion of seminal historical articles and recent studies published up to June 2026. Eligible publications included original experimental studies, review articles, and clinical reports addressing MSC-based or MSC-derived extracellular vesicle-based interventions in Alzheimer's disease, Parkinson's disease, acute CNS injuries such as stroke, and other CNS-related conditions.

The selected literature was then organized into three thematic domains. The first domain addressed the mechanisms of action of MSCs and MSC-derived extracellular vesicles, including paracrine signaling, mitochondrial transfer, efferocytosis, modulation of the glymphatic system, post-transcriptional regulation, and emerging epigenetic mechanisms. The second domain addressed preclinical and clinical evidence of therapeutic application, grouped into five CNS macrocategories according to shared pathophysiological mechanisms and common therapeutic targets: (1) cerebrovascular and ischemic/hemorrhagic disorders; (2) neurodegenerative proteinopathies and related disorders; (3) neurotrauma and acute lesions; (4) autoimmune, inflammatory, and emerging disorders; and (5) retinal diseases as CNS-related sensory disorders. The third domain addressed translational challenges, clinical implications, and future perspectives.

## 3. Mesenchymal Stromal Cells: Sources and Mechanisms

Although MSCs were initially investigated as candidates for tissue replacement, current evidence indicates that their therapeutic effects depend predominantly on context-dependent secretory, immunomodulatory, and intercellular mechanisms rather than on durable engraftment or direct differentiation (Lu and Allickson, 2025[43]; Renesme et al., 2025[61]). Accordingly, this section first summarizes MSC identity and tissue sources (Section 3.1), then discusses the principal paracrine and cellular mechanisms underlying their therapeutic activity (Section 3.2), and finally examines post-transcriptional and epigenetic modulation mediated by MSC-derived extracellular vesicles in recipient neural and immune cells (Section 3.3).

### 3.1. MSC identity and sources

MSCs are multipotent progenitor cells defined by a set of minimum criteria established by the International Society for Cellular Therapy (Dominici et al., 2006[15]). These criteria include: (i) plastic adherence under standard culture conditions; (ii) expression of specific surface markers, including CD73, CD90, and CD105, together with the absence of hematopoietic markers such as CD45 and CD34; and (iii) the capacity for in vitro differentiation into osteogenic, chondrogenic, and adipogenic lineages. Originally isolated from bone marrow (Pittenger et al., 1999[54]), MSCs have since been identified in several adult and perinatal tissues, including adipose tissue, umbilical cord, and placenta. Historically, the acronym MSC has been used to refer to both “mesenchymal stem cells” and “mesenchymal stromal cells.” However, current International Society for Cell & Gene Therapy recommendations support the term “mesenchymal stromal cells” unless stemness has been demonstrated through appropriate functional criteria. This terminology also reflects the growing evidence that the therapeutic effects of MSCs are mediated predominantly by secretory, immunomodulatory, and microenvironmental mechanisms rather than by direct differentiation and long-term tissue replacement (Lu and Allickson, 2025[43]; Renesme et al., 2025[61]; Viswanathan et al., 2019[76]; Wiest, 2025[81]). Accordingly, the term “mesenchymal stromal cells” is used throughout this manuscript.

MSCs can be isolated from various adult and perinatal tissues, and their biological properties may vary according to tissue source, donor characteristics, culture conditions, and manufacturing procedures. Bone marrow-derived MSCs (BM-MSCs) were among the first MSC populations to be characterized, whereas adipose tissue-derived MSCs (AD-MSCs) have attracted considerable interest owing to their relative accessibility and high proliferative capacity. Perinatal sources, including umbilical cord-derived MSCs (UC-MSCs) and placental MSCs, may offer advantages related to lower donor age, reduced senescence-associated changes, and favorable immunomodulatory profiles. In addition, iMSCs have emerged as a potentially scalable and more homogeneous platform that may help reduce donor-to-donor variability, although their clinical translation still requires further validation (Buitrago et al., 2024[7]; Lu and Allickson, 2025[43]).

### 3.2. Paracrine and cellular mechanisms

The therapeutic paradigm of MSCs has shifted from long-term cellular engraftment and direct tissue replacement toward paracrine and immunomodulatory mechanisms (Galipeau and Sensébé, 2018[18]). MSC activity is highly context-dependent and may be modulated by inflammatory signals in the local microenvironment, a process commonly referred to as “licensing.” Upon exposure to pro-inflammatory cytokines, such as interferon-gamma (IFN-γ), tumor necrosis factor-alpha (TNF-α), and interleukin-1 beta (IL-1β), or to ex vivo preconditioning strategies such as hypoxia or three-dimensional culture, MSCs may acquire an activated phenotype characterized by increased secretion of immunomodulatory, trophic, and reparative mediators (Hoseinzadeh et al., 2025[28]; Schepici et al., 2022[64]; Uccelli et al., 2008[74]).

EVs, including exosomes when specifically characterized as such, represent an important component of the MSC secretome. MSC-derived extracellular vesicles (MSC-EVs) can transport proteins, lipids, regulatory microRNAs, and other molecular cargo involved in intercellular communication. In experimental models, MSC-derived EVs have been reported to cross or interact with the blood-brain barrier (BBB), supporting their investigation as cell-free therapeutic candidates for CNS disorders (Guy and Offen, 2020[23]; Liu et al., 2024[40]). However, because EV populations are heterogeneous, the term “exosomes” should be reserved for studies in which this vesicle subtype has been specifically identified, in accordance with current EV nomenclature and reporting recommendations (Welsh et al., 2024[80]).

Beyond soluble factors and EV-mediated signaling, mitochondrial transfer has emerged as another mechanism by which MSCs may support injured neural cells. Under conditions of oxidative stress or metabolic dysfunction, MSCs can transfer functional mitochondria to damaged cells, thereby providing bioenergetic support and promoting cell survival (Mukkala et al., 2023[48]; Nair et al., 2021[49]). This transfer may occur through tunneling nanotubes (TNTs) and has been associated with mitochondrial trafficking regulators such as the mitochondrial Rho GTPase (Miro1). Experimental evidence suggests that modulation of Miro1 expression may affect mitochondrial transfer efficiency and the metabolic recovery of recipient cells, although the relevance of this mechanism in vivo and across different CNS disorders requires further investigation (Bagno et al., 2022[5]; Mukkala et al., 2023[48]).

Emerging evidence also suggests that the immunomodulatory effects of infused MSCs may depend in part on their apoptosis and subsequent clearance by host phagocytes. According to the “dying stem cell hypothesis,” apoptotic MSCs can be engulfed by macrophages through efferocytosis, thereby contributing to macrophage reprogramming toward an anti-inflammatory or reparative phenotype (Harrell and Volarevic, 2021[25]; Hoseinzadeh et al., 2025[28]; Mandl et al., 2025[44]). This process may help induce sustained immunomodulatory responses even after the administered MSCs have cleared, providing a mechanistic explanation for therapeutic effects that are independent of long-term MSC persistence.

In addition to these direct paracrine and cell-mediated mechanisms, MSC-based interventions may influence functional clearance pathways within the CNS. The glymphatic system is an aquaporin-4 (AQP4)-dependent paravascular pathway involved in cerebrospinal fluid-interstitial fluid exchange, metabolic waste clearance, and neuroimmune regulation (Zhao et al., 2026[92]). Aging and acute CNS injuries have been associated with AQP4 disorganization, impaired interstitial fluid clearance, and glymphatic dysfunction. In preclinical models, particularly in intracerebral hemorrhage, AQP4-modified MSCs have been reported to improve glymphatic transport, reduce BBB disruption, and attenuate toll-like receptor 4 (TLR4)/nuclear factor kappa B (NF-κB)-mediated neuroinflammatory signaling (Li et al., 2026[37]).

Accordingly, the key paracrine, cellular, and functional mechanisms underlying the therapeutic actions of MSCs and MSC-derived EVs in the CNS are summarized in Table 1(Tab. 1) (References in Table 1: Amin et al., 2025[3]; An et al., 2025[4]; Bagno et al., 2022[5]; Brooks et al., 2022[6]; Chen et al., 2021[11]; Guy and Offen, 2020[23]; Harrell and Volarevic, 2021[25]; Harrell et al., 2021[24]; Hoseinzadeh et al., 2025[28]; Li et al., 2026[37]; Liu et al., 2024[40]; Mandl et al., 2025[44]; Mukkala et al., 2023[48]; Nair et al., 2021[49]; Nasirishargh et al., 2021[50]; Nazari et al., 2023[51]; Qin et al., 2022[57]; Rather et al., 2023[59]; Shin and Lee, 2020[67]; Vargas-Rodríguez et al., 2023[75]; Welsh et al., 2024[80]; Wu et al., 2020[83]; Xin et al., 2013[85]; Xiong et al., 2024[86]; Yin et al., 2023[88]; Yu et al., 2020[89]; Zayed et al., 2025[90]; Zhao et al., 2026[92]).

### 3.3. Post-transcriptional and epigenetic modulation by MSC-EVs

In addition to transporting trophic and immunomodulatory proteins, MSC-EVs can deliver regulatory RNAs that modify gene expression programs in recipient neural and immune cells. Current evidence indicates that microRNAs (miRNAs) and long non-coding RNAs (lncRNAs) are the best-characterized regulatory components of MSC-EVs. These molecules act predominantly through post-transcriptional mechanisms, including messenger RNA (mRNA) degradation and translational repression, but may also influence epigenetic regulators such as histone-modifying enzymes. However, the available evidence is more robust for post-transcriptional regulation than for direct and stable changes in DNA methylation, histone acetylation, or chromatin accessibility in recipient neural cells (Harrell et al., 2021[24]; Nasirishargh et al., 2021[50]; Volarevic et al., 2025[77]).

At the neuroimmune interface, MSC-EV-associated miRNAs may dampen inflammatory signaling and promote a more reparative cellular environment. Giunti et al. (2021[22]) demonstrated that small EVs derived from IFN-γ-primed MSCs were enriched for miR-467f and miR-466q. Upon transfer to activated microglia, these miRNAs reduced the expression of Map3k8 and Mk2, attenuated p38 mitogen-activated protein kinase (MAPK) signaling, and decreased the expression of pro-inflammatory mediators. MSC-EV-associated miR-146a has also been implicated in regulating the IRAK1/TRAF6/NF-κB signaling axis, thereby contributing to reduced inflammatory activation (Harrell et al., 2021[24]; Kråkenes et al., 2024[32]). In addition, studies using Wharton's jelly MSC-derived small EVs with reduced mature miRNA content suggest that EV-associated miRNAs collectively contribute to decreased microglial activation and improved oligodendrocyte maturation after premature white matter injury (Tscherrig et al., 2024[72]). These findings support the role of MSC-EV cargo in modulating neuroinflammation and creating a more permissive microenvironment for neural repair.

MSC-EV-associated RNAs may also contribute to structural and synaptic plasticity. Seminal experiments demonstrated that MSC-derived exosome-enriched extracellular particles can transfer miR-133b to neural cells, thereby enhancing neurite outgrowth in vitro and promoting neurite remodeling and functional recovery after experimental stroke, in part by downregulating RhoA-related signaling (Xin et al., 2012[84], 2013[85]). Other regulatory miRNAs carried by MSC-EVs have been linked to neuronal survival, axonal growth, and neural differentiation. For example, the miR-17-92 cluster may promote neurite remodeling through PTEN/Akt/mTOR-related pathways, whereas miR-124 may support neuronal differentiation by regulating Sox9 expression (Nasirishargh et al., 2021[50]; Reza-Zaldivar et al., 2018[62]).

Evidence also suggests that MSC-EV cargo can influence histone-regulating pathways. MSC-EV-associated miR-29a has been reported to reduce histone deacetylase 4 (HDAC4) expression and restore the expression of genes related to memory and synaptic plasticity in experimental Alzheimer's disease models (Chen et al., 2021[11]). This finding provides a mechanistic link between MSC-EV-delivered miRNAs and the regulation of histone-modifying enzymes. MSC-EV-delivered lncRNAs may provide an additional layer of gene regulation by acting as competing endogenous RNAs. In a preclinical model of autism spectrum disorder, the lncRNA IFNG-AS1/Ifngas1 acted as a molecular sponge for miR-21a-3p, thereby modulating phosphoinositide 3-kinase/protein kinase B (PI3K/Akt) signaling, supporting neurogenesis, and attenuating inflammatory responses (Fu et al., 2024[17]).

Taken together, the available findings support post-transcriptional regulation mediated by MSC-EV-associated miRNAs and lncRNAs as the most consistently demonstrated mechanism. By contrast, direct effects on DNA methylation, histone acetylation, and chromatin remodeling remain less well established in recipient neural cells and require further mechanistic investigation. The principal post-transcriptional and epigenetic mechanisms mediated by MSC-EVs are summarized in Figure 2(Fig. 2).

## 4. Therapeutic Evidence of MSCs in Central Nervous System Disorders

Building on the mechanisms summarized in Table 1(Tab. 1), this section discusses the therapeutic relevance of MSCs and MSC-EVs across CNS disorders. Rather than considering neurological diseases as isolated entities, the evidence is organized according to shared pathophysiological mechanisms and overlapping therapeutic targets.

Accordingly, the following sections examine MSC-based approaches across five CNS macrocategories: (1) cerebrovascular and ischemic/hemorrhagic disorders; (2) neurodegenerative proteinopathies and related disorders; (3) neurotrauma and acute lesions; (4) autoimmune, inflammatory, and emerging disorders; and (5) retinal diseases as CNS-related sensory disorders (Table 2(Tab. 2); References in Table 2: Ahn et al., 2023[1]; Allegretta et al., 2022[2]; Amin et al., 2025[3]; An et al., 2025[4]; Bagno et al., 2022[5]; Brooks et al., 2022[6]; Calle and Zurita, 2025[8]; Chen et al., 2021[11]; Chen et al., 2023[9]; Chen et al., 2026[10]; Frawley et al., 2024[16]; Harrell et al., 2021[24]; Heris et al., 2022[26]; Kråkenes et al., 2024[32]; León-Moreno et al., 2023[34]; Li et al., 2026[37]; Liu and Jia, 2024[39]; Liu et al., 2022[38]; Liu et al., 2024[40]; London et al., 2013[42]; Mead and Tomarev, 2017[46]; Mukai et al., 2021[47]; Nair et al., 2021[49]; Nasirishargh et al., 2021[50]; Nazari et al., 2023[51]; Norouzi-Barough et al., 2023[52]; Popa-Wagner et al., 2026[55]; Qin et al., 2022[57]; Rather et al., 2023[59]; Regmi et al., 2022[60]; Rinendyaputri et al., 2025[63]; Schepici et al., 2022[64]; Shahror et al., 2020[65]; Sheikhi et al., 2025[66]; Shin and Lee, 2020[67]; Shokati et al., 2021[68]; Soares et al., 2022[69]; Uccelli et al., 2021[73]; Vargas-Rodríguez et al., 2023[75]; Wang et al., 2022[79]; Wilson et al., 2023[82]; Wu et al., 2020[83]; Xin et al., 2013[85]; Xiong et al., 2024[86]; Xu et al., 2025[87]; Yin et al., 2023[88]; Yu et al., 2020;[89] Zayed et al., 2025[90]; Zhang et al., 2020[91]; Zhou et al., 2022[93]).

### 4.1. Cerebrovascular diseases and acute ischemic/hemorrhagic injury

In ischemic stroke, intracerebral hemorrhage (ICH), and ischemia/reperfusion (I/R) injury, MSCs and MSC-EVs have shown therapeutic potential in preclinical models and early clinical studies (Brooks et al., 2022[6]; Liu and Jia, 2024[39]; Rinendyaputri et al., 2025[63]). Their effects appear to depend primarily on modulation of the injured neurovascular microenvironment rather than on direct replacement of lost neural cells. In this context, MSC-based interventions may promote angiogenesis, support endogenous repair processes, and attenuate inflammatory responses (Wang et al., 2022[79]).

A central target in acute cerebrovascular injury is the preservation or restoration of BBB integrity. After ischemic or hemorrhagic injury, increased matrix metalloproteinase-9 (MMP-9) activity contributes to tight junction disruption, vasogenic edema, and leukocyte infiltration (Vargas-Rodríguez et al., 2023[75]; Xu et al., 2025[87]). MSCs and MSC-EVs have been reported to counteract these processes by reducing MMP-9 activity, stabilizing endothelial junctions, and modulating endothelial and inflammatory signaling pathways (Vargas-Rodríguez et al., 2023[75]).

MSC-EVs represent a particularly relevant cell-free strategy in cerebrovascular disorders because of their nanoscale structure and capacity to transfer regulatory molecular cargo to injured neural and vascular cells. In experimental stroke models, MSC-EVs have been reported to cross or interact with the BBB and deliver neuroprotective microRNAs to the ischemic tissue (Guy and Offen, 2020[23]; Xu et al., 2025[87]). Among the regulatory miRNAs delivered by MSC-EVs, miR-133b has been associated with reduced neuronal injury and enhanced neurite remodeling after experimental stroke, supporting the contribution of EV-mediated post-transcriptional regulation to neurovascular repair (Nasirishargh et al., 2021[50]; Xin et al., 2013[85]; Zhou et al., 2022[93]; see Section 3.3). In addition, mitochondrial transfer from MSCs to injured endothelial or neural cells has been proposed as a mechanism that may support cellular bioenergetics and improve the resilience of the neurovascular unit after ischemic injury (Liu and Jia, 2024[39]; Vargas-Rodríguez et al., 2023[75]).

In ICH, secondary brain injury is driven by hematoma expansion, oxidative stress, neuroinflammation, BBB disruption, and perihematomal edema (Calle and Zurita, 2025[8]; Zhou et al., 2022[93]). Recent preclinical evidence has also implicated glymphatic dysfunction in the pathophysiology of ICH. Hemorrhagic injury may disrupt AQP4 organization, impair interstitial fluid clearance, and exacerbate edema and inflammatory damage. In a mouse model of ICH, AQP4-overexpressing MSCs were reported to improve glymphatic transport, reduce BBB disruption, attenuate neuroinflammatory signaling, and improve neurological outcomes (Li et al., 2026[37]). Mechanistically, this study suggested that AQP4 may interact with TLR4, thereby contributing to suppression of downstream NF-κB signaling and reduced inflammatory amplification after hemorrhagic injury (Li et al., 2026[37]).

The immunomodulatory properties of MSCs are also relevant in the inflammatory microenvironment of stroke. MSCs and MSC-EVs may promote a shift in microglia and macrophages from pro-inflammatory phenotypes toward anti-inflammatory or reparative states, accompanied by reduced production of cytokines such as TNF-α and interleukin-6 (IL-6), and increased expression of anti-inflammatory mediators such as interleukin-10 (IL-10) and transforming growth factor-beta (TGF-β) (Liu and Jia, 2024[39]; Wang et al., 2022[79]). This immunomodulatory effect may be particularly relevant in the aging brain, where post-stroke repair is limited by reduced plasticity and a persistent low-grade inflammatory state often referred to as inflammaging. In this context, MSC-EVs may offer advantages as a cell-free strategy capable of delivering regulatory signals to the injured microenvironment while avoiding some of the limitations associated with live-cell administration (Popa-Wagner et al., 2026[55]).

Clinical evidence remains preliminary but generally supports the feasibility and safety of MSC-based approaches in cerebrovascular disease. Early clinical studies and recent reviews suggest that MSC administration through intravenous, intracerebral, or intraventricular routes may be associated with improvements in neurological and functional outcomes, including measures such as the National Institutes of Health Stroke Scale (NIHSS) and the Barthel Index (Brooks et al., 2022[6]; Calle and Zurita, 2025[8]). However, available studies remain limited by small sample sizes, heterogeneity in MSC source and manufacturing procedures, variability in route and timing of administration, and differences in outcome measures. Further well-controlled clinical trials are needed to define optimal cell or EV dose, delivery route, therapeutic window, and preconditioning strategies, including hypoxia or three-dimensional culture priming (Liu and Jia, 2024[39]; Rinendyaputri et al., 2025[63]).

### 4.2. Neurodegenerative proteinopathies and related disorders

Neurodegenerative diseases such as Alzheimer's disease (AD), Parkinson's disease (PD), and amyotrophic lateral sclerosis (ALS) share several convergent pathophysiological features, including progressive neuronal dysfunction, accumulation of misfolded or aggregation-prone proteins, mitochondrial impairment, oxidative stress, synaptic failure, and chronic neuroinflammation (Wilson et al., 2023[82]). In this context, MSCs and MSC-EVs have been investigated not as direct neuronal replacement strategies, but as modulators of the diseased neural microenvironment, with potential effects on immunoregulation, trophic support, mitochondrial homeostasis, and protein clearance (Isik et al., 2025[30]; Regmi et al., 2022[60]; Yin et al., 2023[88]).

In AD, preclinical studies suggest that MSC-EVs may exert neuroprotective and immunomodulatory effects by influencing amyloid-beta (Aβ) metabolism, tau phosphorylation, microglial activation, and synaptic integrity (Chen et al., 2021[11]; Hernández and García, 2021[27]). MSC-EVs have been reported to carry enzymes or regulatory molecules associated with Aβ degradation, including neprilysin (NEP) and insulin-degrading enzyme (IDE), and may contribute to extracellular Aβ clearance in experimental models (Rather et al., 2023[59]; Yin et al., 2023[88]). In addition, EV-associated miRNAs, including miR-29c-3p and miR-146a, have been implicated in regulating amyloidogenic processing and neuroinflammatory responses, including beta-site amyloid precursor protein-cleaving enzyme 1 (BACE1)-associated pathways and microglial activation (Regmi et al., 2022[60]; Wang et al., 2023[78]). MSC-based interventions may also influence autophagy-related pathways, including Beclin-1 and microtubule-associated protein 1A/1B-light chain 3-II (LC3-II) signaling, thereby supporting lysosomal clearance of intracellular protein aggregates and reducing apoptosis-associated signaling by modulating the Bax/Bcl-2 ratio (Qin et al., 2022[57]). Recent bioengineering approaches, including surface modification of MSC-EVs with brain-targeting peptides such as rabies virus glycoprotein (RVG), have been proposed to improve CNS delivery, although their clinical relevance remains to be established (Yin et al., 2023[88]).

In PD, MSCs and MSC-EVs have been investigated for their potential to reduce dopaminergic neuronal vulnerability, oxidative stress, mitochondrial dysfunction, neuroinflammation, and α-synuclein-associated toxicity (Heris et al., 2022[26]; Liu et al., 2022[38]; Volarevic et al., 2025[77]). Recent evidence focused on MSC-EVs suggests that these vesicles may suppress microglial activation, reduce pro-inflammatory cytokine signaling, deliver neuroprotective and immunomodulatory molecular cargo, and support dopaminergic neuron survival in experimental models of PD (Volarevic et al., 2025[77]). Beyond secreting neurotrophic factors such as brain-derived neurotrophic factor (BDNF) and glial cell line-derived neurotrophic factor (GDNF), MSCs may influence the extracellular clearance of α-synuclein. For example, MSC-derived matrix metalloproteinase-2 (MMP-2) has been associated with the degradation of extracellular α-synuclein fibrils, whereas galectin-1 may interfere with mechanisms involved in α-synuclein uptake and intercellular propagation (Heris et al., 2022[26]; Shin and Lee, 2020[67]). In addition, MSC-secreted mediators and MSC-EV cargo may promote microglial polarization toward anti-inflammatory or phagocytic phenotypes, potentially facilitating the clearance of α-synuclein-associated debris and attenuating chronic neuroinflammation (Shin and Lee, 2020[67]; Volarevic et al., 2025[77]).

In ALS, MSC-based approaches have been explored as supportive strategies to protect upper and lower motor neurons through trophic, immunomodulatory, and anti-inflammatory mechanisms (Frawley et al., 2024[16]; Lewis and Suzuki, 2014[36]). Rather than replacing lost motor neurons, MSCs may secrete factors such as vascular endothelial growth factor (VEGF) and GDNF that support motor neuron survival, neuromuscular junction maintenance, and local microenvironmental stability (Frawley et al., 2024[16]). MSCs may also modulate astrocyte and microglial reactivity through TGF-β-related signaling and other immunoregulatory pathways, thereby attenuating non-cell-autonomous mechanisms that contribute to motor neuron degeneration (Frawley et al., 2024[16]). Although early clinical studies using autologous MSCs in ALS have generally supported procedural safety, therapeutic efficacy remains variable, likely reflecting disease heterogeneity, differences in disease stage, MSC source, delivery route, and manufacturing protocols (Frawley et al., 2024[16]).

Beyond disease-specific mechanisms, impaired protein clearance and extracellular fluid homeostasis are increasingly recognized as relevant features of neurodegeneration. The glymphatic system has been implicated in the clearance of Aβ, tau, α-synuclein, and other aggregation-prone proteins, and aging-related disruption of interstitial fluid dynamics may contribute to their accumulation (Zhao et al., 2026[92]). In this context, MSC-based interventions may influence glymphatic-related clearance indirectly through effects on astrocytic AQP4 organization, neuroinflammation, vascular integrity, and interstitial fluid drainage. In Huntington's disease models, MSC administration has been associated with improved AQP4-M23 expression, reduced astrogliosis, and enhanced distribution of antisense oligonucleotides (ASOs), suggesting a potential interaction between MSC therapy, glymphatic function, and delivery of disease-modifying therapeutics (Wu et al., 2020[83]). Similarly, the combination of adipose tissue-derived MSCs with pharmacological glymphatic modulation has been reported to improve clearance of pathogenic prion protein and survival in preclinical prion disease models (Zayed et al., 2025[90]). These findings support the hypothesis that MSC-based approaches may contribute to neurodegenerative disease management not only by modulating inflammation and trophic support but also by improving functional clearance pathways in selected experimental contexts.

### 4.3. Neurotrauma and acute lesions

Acute CNS injuries, including traumatic brain injury (TBI), spinal cord injury (SCI), hypoxic-ischemic encephalopathy (HIE), and intraventricular hemorrhage (IVH), are characterized by primary mechanical, ischemic, or hemorrhagic damage followed by secondary injury cascades. These include mitochondrial dysfunction, oxidative stress, BBB disruption, excitotoxicity, neuroinflammation, glial activation, and progressive cell death (Ahn et al., 2023[1]; Nair et al., 2021[49]). In these contexts, MSCs and MSC-EVs have been investigated as modulators of the injured CNS microenvironment, with potential effects on mitochondrial preservation, inflammatory regulation, vascular stabilization, and endogenous repair.

In perinatal brain injuries, the immature brain is particularly vulnerable to oxidative damage because of limited antioxidant capacity and high metabolic demand. MSCs may attenuate this vulnerability by supporting mitochondrial homeostasis, including the transfer of functional mitochondria to injured neural cells through TNTs (Nair et al., 2021[49]). In addition, MSC-based interventions have been associated with improved mitochondrial quality control and bioenergetic recovery, involving modulation of mitophagy-related pathways and PI3K/Akt/mechanistic target of rapamycin (mTOR)-associated survival signaling (Nair et al., 2021[49]). In severe neonatal IVH, the delivery route appears to be a critical determinant of therapeutic response. Preclinical and early clinical evidence suggest that local intracerebroventricular administration may offer advantages over systemic delivery in selected neonatal injury contexts, with phase I studies supporting the feasibility and preliminary safety of this approach (Ahn et al., 2023[1]).

In adult TBI, MSC-EVs have been investigated as cell-free therapeutic candidates capable of modulating neuroinflammation, edema, BBB integrity, and cell survival. Experimental studies, including large-animal models, suggest that MSC-EVs may reduce cerebral edema, support vascular and junctional stability, and attenuate apoptosis after brain injury (Xiong et al., 2024[86]). Their therapeutic effects appear to be partly mediated by regulatory microRNA cargo. For example, MSC-EVs enriched with the miR-17-92 cluster have been reported to enhance neurogenesis, angiogenesis, and functional recovery following experimental traumatic brain injury, illustrating the relevance of EV-mediated delivery of regulatory RNAs in post-traumatic repair (Xiong et al., 2024[86]; see Section 3.3).

Repetitive traumatic brain injury (rTBI) may also impair glymphatic and meningeal lymphatic function, contributing to the accumulation of acetylated tau (ac-tau), axonal injury, and cognitive dysfunction. In this setting, engineered MSC-EVs enriched with nicotinamide phosphoribosyltransferase (NAMPT) have been reported to attenuate neuroinflammation, preserve AQP4 organization, and support meningeal lymphatic and glymphatic clearance in experimental models (Chen et al., 2026[10] . These findings suggest that selected MSC-EV engineering strategies may improve functional clearance pathways after neurotrauma, although further validation is required before clinical translation.

To increase MSC potency in the inflammatory and metabolically compromised post-injury microenvironment, several preconditioning and engineering strategies have been explored. Hypoxic preconditioning may enhance the expression of hypoxia-inducible factor 1-alpha (HIF-1α) and increase the secretion of trophic and proangiogenic mediators. Chemical priming with thrombin has been reported to enhance the paracrine potency of MSCs, increasing EV production and enriching reparative cargo, including factors such as VEGF and BDNF (Ahn et al., 2023[1]; Schepici et al., 2022[64]). Genetic modification has been used to improve MSC homing and immunomodulatory capacity. For example, overexpression of C-X-C chemokine receptor type 4 (CXCR4) may enhance MSC migration toward stromal cell-derived factor 1 (SDF-1), a chemoattractant upregulated at sites of CNS injury (Shahror et al., 2020[65]). Similarly, MSCs engineered to overexpress IL-10 have been reported to promote anti-inflammatory microglial responses and improve functional recovery in experimental neurotrauma models (Nair et al., 2021[49]; Shahror et al., 2020[65]).

In SCI, secondary injury is driven by Wallerian degeneration, oxidative stress, glial scar formation, inflammatory cell infiltration, and activation of inflammasome-dependent cell death pathways (Chen et al., 2023[9]; Nazari et al., 2023[51]). MSC-based therapies may attenuate these processes by promoting macrophage and microglial polarization toward reparative phenotypes, supporting axonal preservation, and modulating the inflammatory milieu (Chen et al., 2023[9]). Recent studies also suggest that MSCs and MSC-EVs can suppress activation of the NOD-, LRR- and pyrin domain-containing protein 3 (NLRP3) inflammasome, thereby reducing caspase-1 activation, IL-1β and interleukin-18 (IL-18) release, and pyroptosis in injured spinal cord tissue (Nazari et al., 2023[51]). Together with autophagy-related clearance of cellular debris, these effects may contribute to reduced secondary tissue damage and improved motor recovery in preclinical SCI models (Chen et al., 2023[9]; Nazari et al., 2023[51]).

### 4.4. Autoimmune, inflammatory, and emerging disorders: Multiple sclerosis, SMI, and Long COVID

Autoimmune, inflammatory, and emerging post-infectious CNS disorders share several pathophysiological features, including persistent neuroinflammation, glial activation, immune dysregulation, BBB disruption, and altered neural network function. In this context, MSCs and MSC-EVs have been investigated as potential modulators of neuroimmune homeostasis rather than as direct cell replacement strategies.

In multiple sclerosis (MS), MSC-based approaches have been primarily explored for their immunomodulatory and pro-repair properties. MSCs and MSC-EVs may contribute to the regulation of adaptive immunity by promoting CD4+ regulatory T-cell (Treg) responses and reducing pro-inflammatory T helper 1 (Th1) and T helper 17 (Th17) activity (Amin et al., 2025[3]; Shokati et al., 2021[68]). In addition, MSCs may influence humoral immunity by modulating B-cell activation, maturation, and autoantibody production, which are relevant to myelin-directed immune responses (Sheikhi et al., 2025[66]). Within the CNS, MSC-EVs enriched with specific regulatory microRNAs, including miR-216a-5p and miR-467f, have been reported to promote microglial polarization toward anti-inflammatory or reparative phenotypes through modulation of TLR4/NF-κB- and PI3K/Akt-related inflammatory pathways (Allegretta et al., 2022[2]; Kråkenes et al., 2024[32]). This shift may facilitate myelin debris clearance and create a permissive environment for oligodendrocyte precursor cell (OPC) maturation and remyelination (Kråkenes et al., 2024[32]). Clinical studies have generally supported the feasibility and safety of intrathecal or intravenous MSC administration in MS, with variable effects on clinical disability and magnetic resonance imaging (MRI) inflammatory activity. However, clinical efficacy remains inconsistent, and larger controlled trials are required to define the optimal cell source, route of administration, dose, timing, and patient selection criteria (Amin et al., 2025[3]; Sheikhi et al., 2025[66]; Shokati et al., 2021[68]; Uccelli et al., 2021[73]).

In the neuropsychiatric field, particularly in SMI such as schizophrenia and bipolar disorder, MSC-based strategies remain investigational. These disorders have been associated with neuroinflammation, synaptic dysconnectivity, oligodendroglial dysfunction, and impaired neural plasticity (Zhang et al., 2020[91]). Experimental evidence suggests that MSCs and MSC-EVs may influence mechanisms relevant to these processes, including immunomodulatory signaling, trophic support, and oligodendrocyte lineage regulation (Bagno et al., 2022[5]; Mukai et al., 2021[47]; Soares et al., 2022[69]). For example, MSC-derived mediators such as indoleamine 2,3-dioxygenase (IDO), prostaglandin E2 (PGE2), and neurotrophic factors may contribute to an anti-inflammatory and pro-repair microenvironment, potentially supporting synaptic and myelin-related plasticity. However, the application of MSC-based interventions to SMI remains at an early conceptual and preclinical stage. Proposed strategies, including cell preconditioning, intranasal delivery, and functional magnetic resonance imaging (fMRI)-guided targeting of disconnected neural circuits such as the default mode network (DMN), should therefore be interpreted as future translational possibilities rather than established therapeutic approaches (Schepici et al., 2022[64]; Zhang et al., 2020[91]).

Long COVID has emerged as a post-infectious condition that may involve persistent systemic inflammation, endothelial dysfunction, BBB disruption, microglial activation, and cognitive symptoms commonly described as “brain fog” (Davis et al., 2023[14]; León-Moreno et al., 2023[34]). Although their mechanisms remain incompletely understood and heterogeneous among patients, MSC-EVs have been proposed as a potential cell-free strategy to modulate the inflammatory and vascular components of post-viral CNS dysfunction. Compared with live-cell administration, MSC-EVs may offer theoretical advantages, including lower risk of pulmonary entrapment and the ability to deliver regulatory molecular cargo through less invasive routes, such as intranasal administration (León-Moreno et al., 2023[34]; Norouzi-Barough et al., 2023[52]). Experimental and early translational approaches have explored EV-based formulations, hydrogels, and nanoparticle-assisted delivery systems to enhance nose-to-brain transport and prolong local therapeutic effects. Nevertheless, evidence supporting MSC-EV-based interventions for Long COVID-related neurological manifestations remains preliminary, and rigorous preclinical and clinical studies are needed before therapeutic conclusions can be drawn (León-Moreno et al., 2023[34]; Norouzi-Barough et al., 2023[52]).

### 4.5. Retinal diseases as CNS-related sensory disorders

The retina is developmentally and anatomically derived from the CNS and shares several cellular, vascular, and neuroinflammatory features with other CNS tissues (London et al., 2013[42]). Retinal diseases, including diabetic retinopathy, age-related macular degeneration, glaucoma, and other retinal degenerative disorders, are characterized by oxidative stress, vascular dysfunction, neuroinflammation, mitochondrial impairment, blood-retinal barrier (BRB) disruption, and progressive loss of photoreceptors or retinal ganglion cells (An et al., 2025[4]; Yu et al., 2020[89]). These shared pathophysiological features support the inclusion of retinal disorders as CNS-related sensory conditions in the context of MSC-based neuroprotective strategies.

MSC-EVs can deliver regulatory miRNAs and other molecular cargo that modulate inflammatory and cell-survival pathways in retinal cells. These mechanisms may attenuate NF-κB- and inflammasome-related signaling, reduce inflammatory activation, and support the survival of retinal ganglion cells and other vulnerable retinal populations (An et al., 2025[4]; Harrell et al., 2021[24]; Mead and Tomarev, 2017[46]; Nasirishargh et al., 2021[50]; see Section 3.3). Through these mechanisms, MSC-EVs may contribute to reduced inflammatory activation, improved retinal cell survival, and preservation of retinal structure in experimental models.

In addition to EV-mediated signaling, MSCs may support retinal bioenergetics through mitochondrial transfer. Experimental evidence suggests that MSCs can donate functional mitochondria to metabolically stressed retinal cells via TNTs, potentially improving mitochondrial function and reducing oxidative injury (An et al., 2025[4]). Although these findings support the therapeutic potential of MSC-based and MSC-EV-based strategies in retinal degeneration, most evidence remains preclinical. Further studies are needed to define optimal vesicle characterization, delivery route, dosing, treatment timing, long-term safety, and functional efficacy before clinical translation.

Table 2(Tab. 2) summarizes the main pathophysiological targets, proposed MSC- and MSC-EV-mediated effects, evidence base, and translational challenges across the five CNS macrocategories.

## 5. Translational Challenges: Safety, Manufacturing, and Regulatory Updates

A critical evaluation of the current literature reveals a substantial translational gap between preclinical promise and clinical efficacy. Although animal studies support the neuroprotective, immunomodulatory, and plasticity-enhancing potential of MSCs, clinical efficacy remains unproven. Early-phase human studies have generally supported short-term safety and feasibility, with some reporting exploratory clinical, imaging, or biomarker signals (Ghasemi et al., 2023[19]; Isik et al., 2025[30]; Lu and Allickson, 2025[43]). A recent randomized phase 2a trial of laromestrocel in mild Alzheimer's disease reported indications of reduced brain atrophy and possible clinical benefit at 39 weeks. However, its small sample size, safety-focused primary endpoint, and exploratory analyses of efficacy preclude conclusions regarding disease modification (Rash et al., 2025[58]). More broadly, no adequately powered confirmatory trial has established a durable disease-modifying effect of MSC-based therapy in neurodegenerative disorders. This gap may reflect the limited ability of experimental models to reproduce the chronic and heterogeneous nature of human neurodegeneration, as well as small cohort sizes, short follow-up periods, and variability in cell sources, doses, delivery routes, and outcome measures. Limited cell persistence and target-tissue exposure may also constrain efficacy, particularly after intravenous administration, although systemic paracrine and immunomodulatory effects may still occur (Bagno et al., 2022[5]; Ghasemi et al., 2023[19]; León-Moreno et al., 2023[34]).

Beyond physiological and biodistribution barriers, the systemic administration of live MSCs presents specific safety and regulatory concerns. Culture-expanded MSCs may express variable levels of tissue factor, which can activate coagulation pathways and contribute to the instant blood-mediated inflammatory reaction, increasing the risk of thromboinflammatory events in susceptible settings (León-Moreno et al., 2023[34]; Lu and Allickson, 2025[43]). Other safety concerns include the potential for ectopic tissue formation, unwanted pro-fibrotic or pro-angiogenic effects, immune incompatibility, and the theoretical risk of malignant transformation after prolonged ex vivo expansion (Guy and Offen, 2020[23]; Lu and Allickson, 2025[43]).

Clinical efficacy assessment is further complicated by inadequate or insufficiently standardized potency assays, which may not adequately reflect the proposed mechanism of action or predict relevant biological activity (Lepski and Arévalo, 2026[35]; Lu and Allickson, 2025[43]; Renesme et al., 2025[61]). This limitation is compounded by insufficient patient stratification according to disease stage and biological phenotype. Clinical trials often include heterogeneous patient populations without stratification based on the stage of neurodegeneration, inflammatory or molecular biomarkers, or other predictors of therapeutic response. Variability in dose, treatment timing, and therapeutic windows may further obscure signals of efficacy across studies (Ghasemi et al., 2023[19]; Lepski and Arévalo, 2026[35]; Lu and Allickson, 2025[43]).

Manufacturing and scalability represent additional barriers to clinical implementation. MSC properties are influenced by donor-to-donor variability, tissue source, donor age, culture media, oxygen tension, passage number, cryopreservation, thawing procedures, and release criteria (An et al., 2025[4]; Lu and Allickson, 2025[43]). GMP-compliant production requires rigorous control of these variables to preserve cell identity, viability, sterility, potency, and batch-to-batch consistency. Extensive passaging may induce senescence and reduce immunomodulatory or trophic activity, whereas cryopreservation and thawing can transiently impair MSC fitness and functional responses (Giovannelli et al., 2023[21]; Lu and Allickson, 2025[43]; Mandl et al., 2025[44]). Accordingly, release testing should include standardized, mechanism-relevant potency assays to support product comparability and regulatory evaluation.

The heterogeneity of clinical outcomes in MSC trials has also emphasized the need for harmonized reporting and regulatory standards. The 2025 ISCT Delphi-based consensus definition and clinical reporting guidelines provide an important framework for improving transparency and reproducibility in MSC-based therapeutics (Renesme et al., 2025[61]; Wiest, 2025[81]). These recommendations emphasize detailed reporting of tissue source, donor characteristics, culture conditions, manufacturing procedures, product characterization, viability at the time of administration, dose, route of delivery, timing, and functional potency testing (Lu and Allickson, 2025[43]; Renesme et al., 2025[61]; Wiest, 2025[81]). Adoption of these standards is particularly relevant for CNS applications, in which differences in delivery route, disease stage, inflammatory status, and outcome measures can substantially influence therapeutic responses.

In this context, MSC-EVs have emerged as a promising cell-free alternative that may reduce some risks associated with live-cell administration. Because MSC-EVs are acellular and non-replicative, they are not expected to engraft, proliferate, or differentiate ectopically. They may also have a lower risk of pulmonary entrapment and offer practical advantages in storage, engineering, dosing, and product standardization (Allegretta et al., 2022[2]; Giovannelli et al., 2023[21]; Guy and Offen, 2020[23]). However, MSC-EV translation faces its own challenges, including vesicle heterogeneity, isolation and purification methods, cargo characterization, potency assays, biodistribution, dosing, scalability, and long-term safety (An et al., 2025[4]; Gimona et al., 2021[20]; Giovannelli et al., 2023[21]; Welsh et al., 2024[80]). Thus, MSC-EVs should not be viewed simply as risk-free substitutes for MSCs, but as distinct biological products requiring dedicated manufacturing and regulatory frameworks.

Building on the therapeutic potential of natural MSC-EVs, bioengineering strategies are being developed to improve targeting, cargo definition, and disease-specific activity. Natural MSC-EVs carry a heterogeneous endogenous cargo of regulatory RNAs, proteins, and lipids, whose composition varies according to MSC source and culture conditions. By contrast, engineered MSC-EVs can be modified at both the surface and cargo levels to improve spatial and molecular precision. Surface modifications incorporating RVG-derived peptides have been investigated to enhance brain accumulation and neural-cell targeting in preclinical models (Cui et al., 2019[13]; Huang et al., 2026[29]; Yin et al., 2023[88]). Cargo-engineering strategies may include enrichment with miR-133b to support neurite remodeling and neuroplasticity, loading with small interfering RNAs (siRNAs) directed against SNCA mRNA to reduce α-synuclein expression, or enrichment with NAMPT to provide metabolic support through nicotinamide adenine dinucleotide (NAD+)/sirtuin 1 (SIRT1)-related pathways (Chen et al., 2026[10]; Cooper et al., 2014[12]; Huang et al., 2026[29]; Isik et al., 2025[30]; Xin et al., 2013[85]). Although the α-synuclein-targeting study used engineered EVs derived from dendritic cells rather than MSCs, it provides proof of concept for RVG-mediated delivery of therapeutic siRNA to the CNS (Cooper et al., 2014[12]). Representative routes considered for CNS delivery include intranasal, intravenous, and intracerebroventricular administration, which differ in invasiveness, biodistribution, and target-tissue exposure (Huang et al., 2026[29]; Isik et al., 2025[30]; Kodali et al., 2023[31]). This transition from natural MSC-EVs with heterogeneous endogenous cargo to precision-engineered MSC-EVs is summarized in Figure 3(Fig. 3).

These approaches may enhance the precision of MSC-EV-based therapies for CNS disorders, but they also introduce additional regulatory complexity regarding product identity, reproducibility, off-target effects, immunogenicity, and safety. Accordingly, engineered MSC-EVs will require product-specific assessments of targeting efficiency, cargo stability, biodistribution, off-target effects, immunogenicity, and long-term safety prior to clinical translation.

## 6. Conclusion and Future Perspectives

MSCs and MSC-EVs represent promising investigational approaches in regenerative neurology and CNS repair. Current evidence indicates that their therapeutic potential is mediated predominantly by paracrine and immunomodulatory mechanisms, including regulation of neuroinflammation, trophic support, modulation of mitochondrial bioenergetics, BBB stabilization, and potential effects on protein and interstitial fluid clearance. Collectively, these mechanisms target convergent pathological processes underlying CNS injury and degeneration, particularly neuroinflammation, barrier dysfunction, and impaired neuroplasticity. Although durable disease-modifying efficacy in humans has not yet been established, the convergence of mechanistic and preclinical evidence provides a strong rationale for continued translational investigation.

Clinical translation will require a cohesive roadmap that links GMP-compliant production and mechanism-relevant potency testing to transparent clinical reporting. GMP procedures should standardize cell sourcing, expansion, cryopreservation, batch consistency, and release criteria for identity, sterility, viability, genomic stability, and potency (Lu and Allickson, 2025[43]). The 2025 ISCT Delphi-based 33-item reporting framework complements these requirements by covering essential products, manufacturing, administration, functional assay, and patient-related variables (Renesme et al., 2025[61]; Wiest, 2025[81]). Together, these measures may improve product comparability, trial reproducibility, and regulatory evaluation, while MSC-EV products will require analogous product-specific standards for isolation, characterization, potency, and release.

MSC-EVs may reduce some limitations associated with live-cell administration, whereas engineered EVs offer opportunities for more targeted and controllable delivery of regulatory cargo. Their development should be supported by rigorous evaluation of biodistribution, product consistency, off-target effects, immunogenicity, and long-term safety. Future studies combining mechanistically informed preclinical models with adequately powered and appropriately stratified clinical trials will be essential to define disease-specific indications, therapeutic windows, and clinically meaningful outcomes. With improved standardization and precision engineering, MSC- and MSC-EV-based strategies may advance toward more reproducible and personalized approaches for CNS repair.

## Declaration

### Author contributions

S.K.S. led the writing of the original draft and contributed to conceptualization, methodology, and investigation. I.V.R.S. and J.S.L.L. contributed to conceptualization, methodology, investigation, and writing - original draft. L.L.S.M. contributed to conceptualization, methodology, supervision, project administration, and writing - review and editing. All authors reviewed and edited the manuscript, approved the final version of the manuscript, and agreed to be accountable for the accuracy and integrity of the work.

### Artificial Intelligence (AI) - assisted technologies

The authors used AI-assisted technologies in preparing this manuscript. Large Language Model-based tools, including NotebookLM by Google and ChatGPT by OpenAI, were used to improve language, readability, clarity, and editorial consistency. Gemini by Google was used to generate initial versions of Figures 1(Fig. 1) and 2(Fig. 2), whereas ChatGPT by OpenAI was used to generate the initial version of Figure 3(Fig. 3). The authors subsequently reviewed all generated visual elements for scientific accuracy and edited them in Adobe Photoshop. After using these tools, the authors carefully reviewed and edited the content as needed. The authors take full responsibility for the content, accuracy, and integrity of the submitted work.

### Conflict of interest

The authors disclose no conflicts of interest.

### Acknowledgments

The authors acknowledge the Specialization Course in Neuroscience at Universidade Federal de São Paulo (UNIFESP) for providing the academic context that motivated this manuscript. This study was financed in part by the Coordenação de Aperfeiçoamento de Pessoal de Nível Superior - Brasil (CAPES) - Finance Code 001.

## Figures and Tables

**Table 1:**
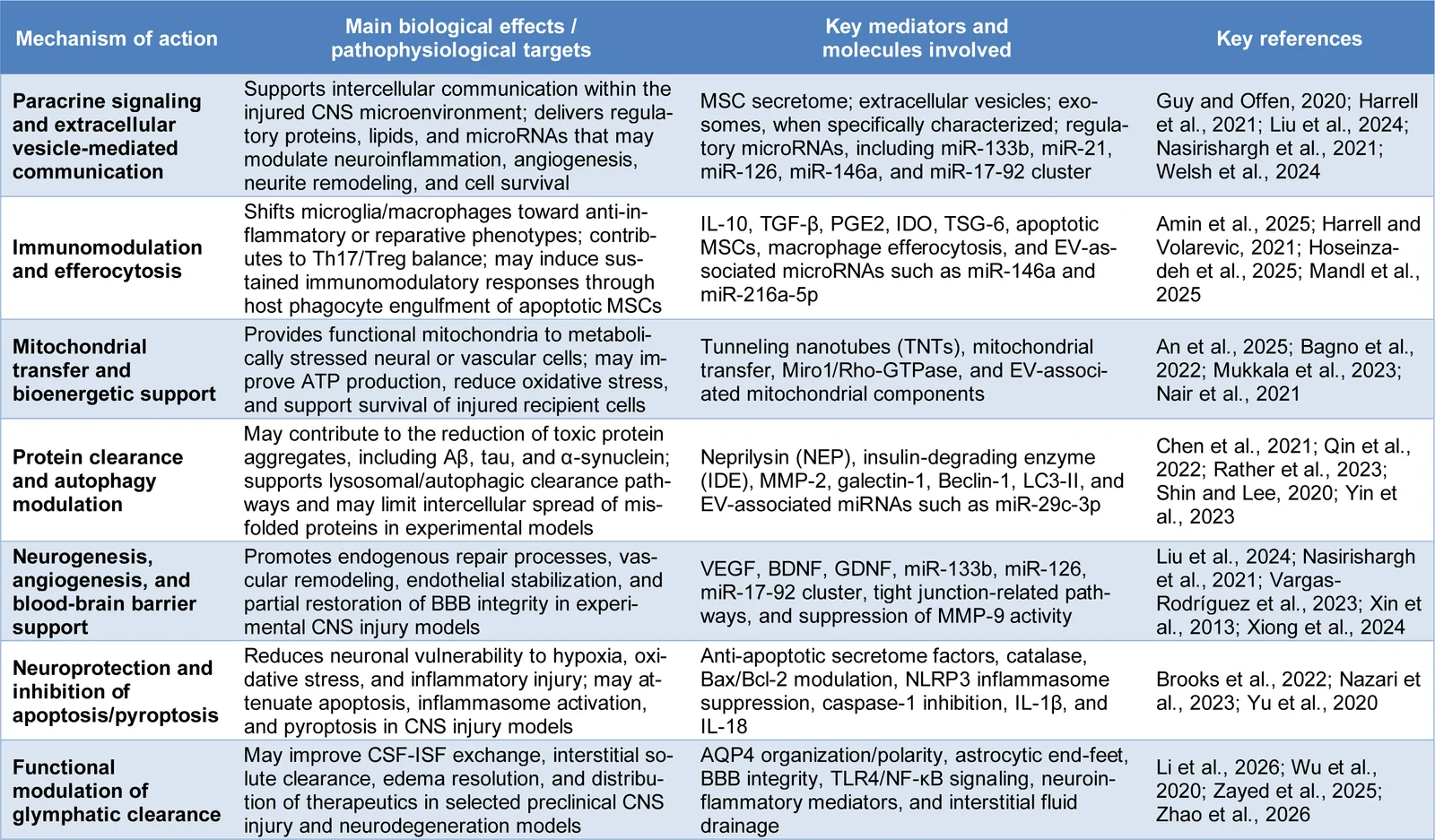
Key paracrine and cellular mechanisms underlying MSC and MSC-EV therapeutic actions in the Central Nervous System (CNS)

**Table 2:**
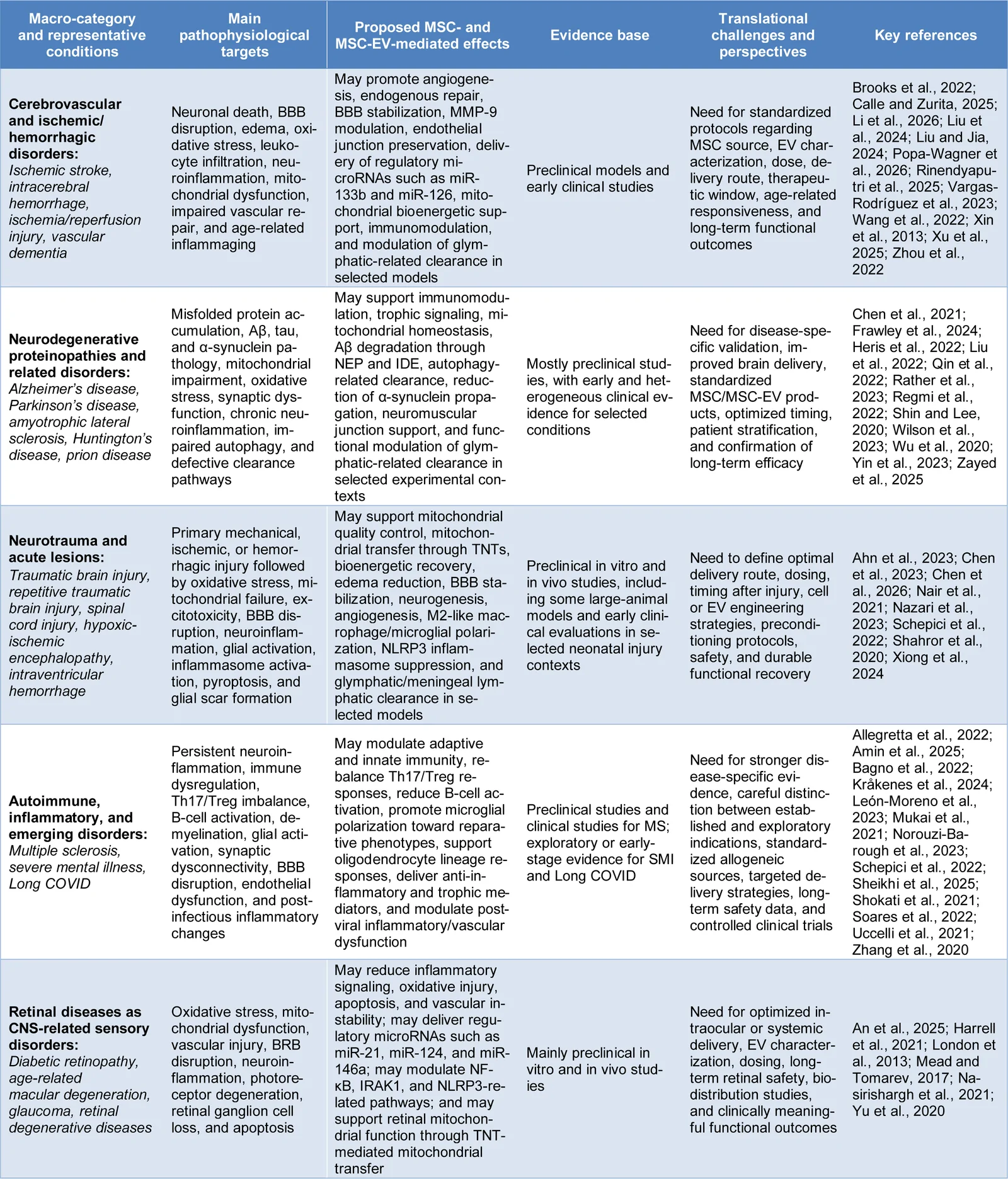
Pathophysiological targets, proposed MSC- and MSC-EV-mediated effects, evidence base, and translational challenges across central nervous system disorder macro-categories

**Figure 1:**
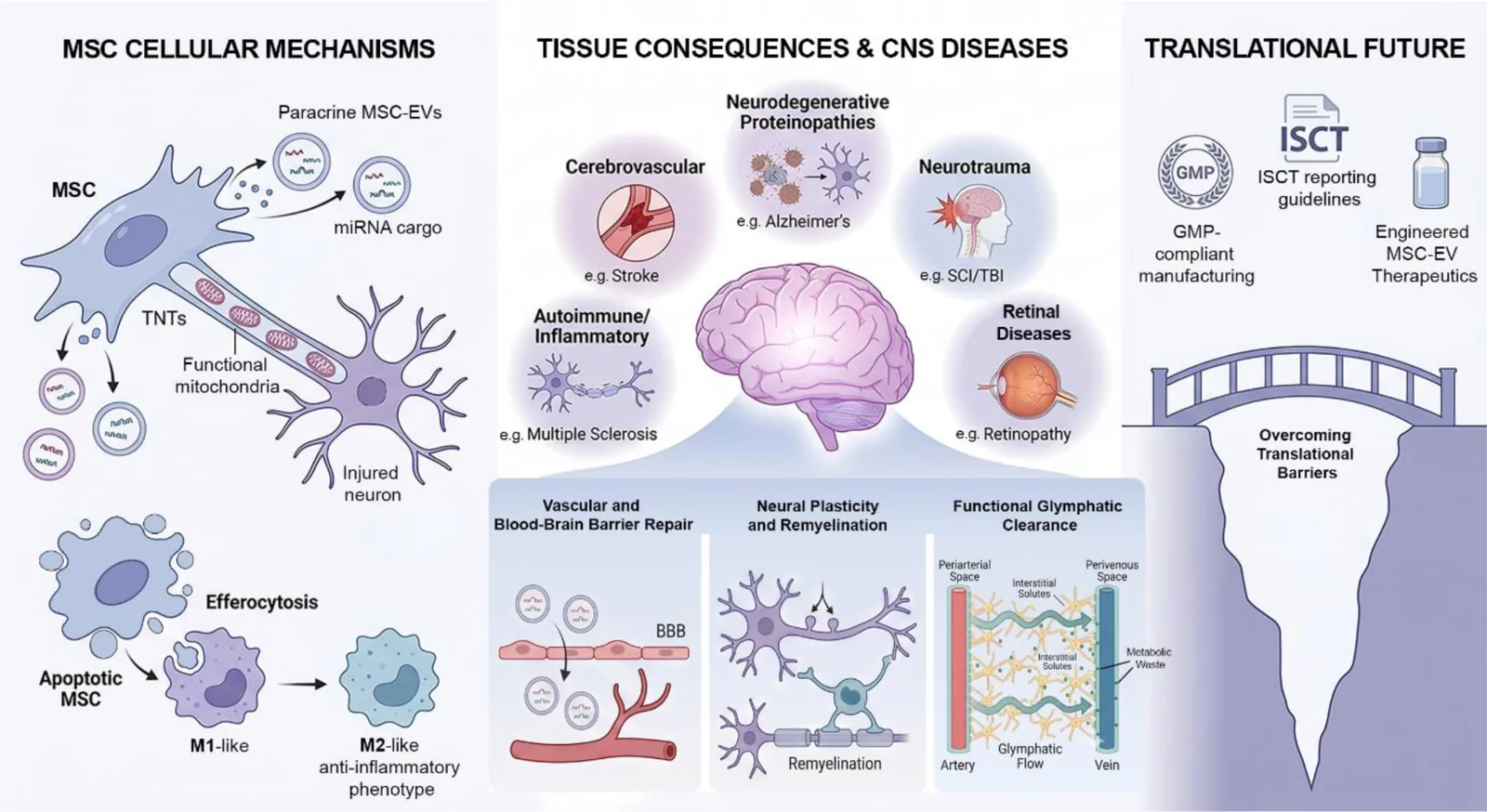
Graphical abstract: Mesenchymal stromal cell-mediated paracrine modulation of neuroplasticity in the central nervous system. On the left, mesenchymal stromal cells (MSCs) are represented as modulators of the neural microenvironment through MSC-derived extracellular vesicles (MSC-EVs), miRNA cargo delivery, mitochondrial transfer via tunneling nanotubes (TNTs), and efferocytosis of apoptotic MSCs by host phagocytes, favoring anti-inflammatory and reparative responses. In the center, these mechanisms are linked to major categories of central nervous system (CNS) disorders, including cerebrovascular and ischemic/hemorrhagic disorders, neurodegenerative proteinopathies, neurotrauma, autoimmune/inflammatory conditions, and retinal diseases, which are classified as CNS-related sensory disorders. Downstream tissue-level effects include vascular and blood-brain barrier (BBB) repair, neural plasticity and remyelination, and functional glymphatic clearance. On the right, the translational pathway is represented by the need to overcome barriers related to GMP-compliant manufacturing, ISCT reporting guidelines, and the development of engineered MSC-EV-based cell-free therapeutic strategies. The graphical abstract was created using the AI-assisted image generation tool Gemini and subsequently edited and scientifically reviewed by the authors in Adobe Photoshop. Abbreviations: BBB, blood-brain barrier; CNS, central nervous system; CSF, cerebrospinal fluid; GMP, Good Manufacturing Practice; ISCT, International Society for Cell & Gene Therapy; ISF, interstitial fluid; miRNA, microRNA; MSC, mesenchymal stromal cell; MSC-EVs, mesenchymal stromal cell-derived extracellular vesicles; TNTs, tunneling nanotubes.

**Figure 2:**
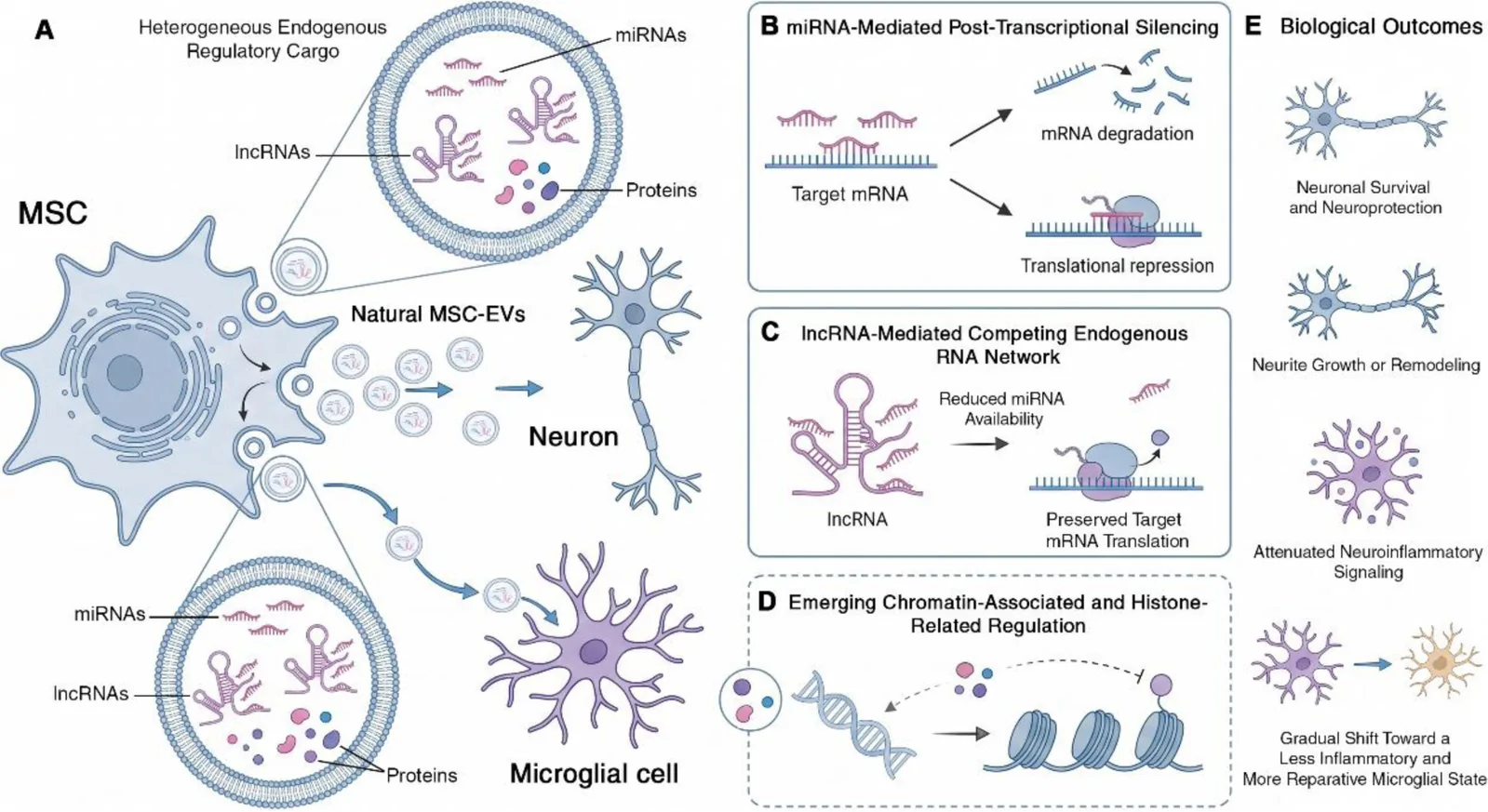
Post-transcriptional and epigenetic modulation mediated by mesenchymal stromal cell-derived extracellular vesicles. (A) Natural mesenchymal stromal cell-derived extracellular vesicles (MSC-EVs) released by MSCs carry heterogeneous endogenous regulatory cargo, including microRNAs (miRNAs), long non-coding RNAs (lncRNAs), and proteins, and interact with recipient neurons and microglial cells. (B) Following cellular uptake, miRNAs regulate gene expression primarily through post-transcriptional mechanisms by promoting target messenger RNA (mRNA) degradation or translational repression. (C) In parallel, lncRNAs may function within competing endogenous RNA (ceRNA) networks by binding and sequestering miRNAs, thereby modifying the availability of these molecules to regulate their target transcripts. (D) Emerging evidence also suggests that MSC-EV cargo may indirectly influence chromatin-associated pathways and histone-related regulatory mechanisms, although evidence for direct and stable changes in DNA methylation, histone acetylation, or chromatin accessibility in recipient neural cells remains less well established than evidence for post-transcriptional regulation. (E) Collectively, these mechanisms may support neuronal survival and neuroprotection, neurite growth or remodeling, attenuation of neuroinflammatory signaling, and a gradual shift toward a less inflammatory and more reparative microglial state. The figure was created using the AI-assisted image generation tool Gemini and subsequently reviewed for scientific accuracy and edited by the authors using Adobe Photoshop. Abbreviations: ceRNA, competing endogenous RNA; lncRNA, long non-coding RNA; miRNA, microRNA; mRNA, messenger RNA; MSC, mesenchymal stromal cell; MSC-EVs, mesenchymal stromal cell-derived extracellular vesicles.

**Figure 3:**
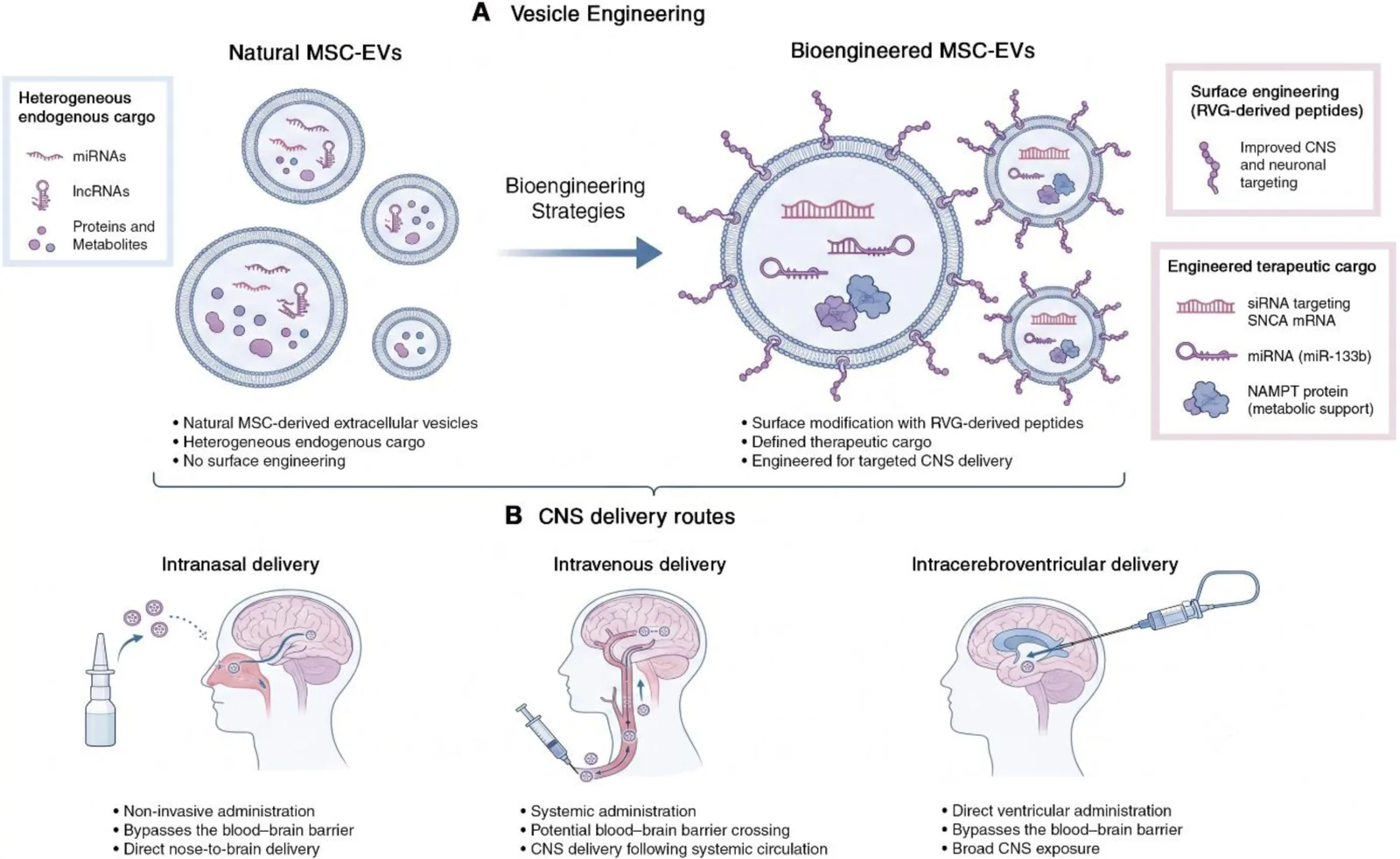
Transition from natural to bioengineered mesenchymal stromal cell-derived extracellular vesicles for precision central nervous system therapy. (A) Natural mesenchymal stromal cell-derived extracellular vesicles (MSC-EVs), carrying heterogeneous endogenous cargo, are contrasted with bioengineered MSC-EVs containing defined therapeutic cargo and surface modifications designed to improve central nervous system (CNS) targeting. Surface engineering with rabies virus glycoprotein (RVG)-derived peptides has been investigated in preclinical models to enhance brain accumulation and neuronal targeting. Representative engineered cargo includes microRNA-133b (miR-133b) to support neurite remodeling and neuroplasticity, small interfering RNA (siRNA) directed against SNCA mRNA to reduce α-synuclein expression, and nicotinamide phosphoribosyltransferase (NAMPT) to support NAD+/SIRT1-related metabolic pathways. The SNCA-targeting siRNA example is presented as proof of concept because the corresponding experimental study used extracellular vesicles derived from dendritic cells rather than MSCs. (B) Representative CNS delivery routes include intranasal, intravenous, and intracerebroventricular administration, illustrating different strategies for delivering natural or engineered MSC-EVs to the CNS, which differ in invasiveness, biodistribution, and target-tissue exposure. Together, these bioengineering strategies aim to improve molecular precision, CNS targeting, and therapeutic specificity compared with natural MSC-EVs. The figure was created using the AI-assisted image generation tool ChatGPT and subsequently reviewed for scientific accuracy and edited by the authors using Adobe Photoshop. Abbreviations: CNS, central nervous system; miR-133b, microRNA-133b; MSC, mesenchymal stromal cell; MSC-EVs, mesenchymal stromal cell-derived extracellular vesicles; NAD+, nicotinamide adenine dinucleotide; NAMPT, nicotinamide phosphoribosyltransferase; RVG, rabies virus glycoprotein; dbchsiRNA, small interfering RNA; SNCA, synuclein alpha gene; SIRT1, sirtuin 1.
